# Cell-cell communication characteristics in breast cancer metastasis

**DOI:** 10.1186/s12964-023-01418-4

**Published:** 2024-01-19

**Authors:** Jingtong Xu, Fangyan Gao, Weici Liu, Xiaoxiang Guan

**Affiliations:** 1 Department of Oncology, The First Affiliated Hospital of Nanjing Medical University, Nanjing, 210029 China; 2 The Affiliated Wuxi People’s Hospital of Nanjing Medical University, Wuxi People’s Hospital, Wuxi Medical Center, Nanjing Medical University, Wuxi, 214023 Jiangsu China; 3 Jiangsu Key Lab of Cancer Biomarkers, Prevention and Treatment, Collaborative Innovation Center for Personalized Cancer Medicine, Nanjing Medical University, Nanjing, 210029 China

**Keywords:** Breast cancer, Metastasis, Microenvironment, Communications, Crosstalk

## Abstract

Breast cancer, a highly fatal disease due to its tendency to metastasize, is the most prevalent form of malignant tumors among women worldwide. Numerous studies indicate that breast cancer exhibits a unique predilection for metastasis to specific organs including the bone, liver, lung, and brain. However, different types of, The understanding of the heterogeneity of metastatic breast cancer has notably improved with the recent advances in high-throughput sequencing techniques. Focusing on the modification in the microenvironment of the metastatic organs and the crosstalk between tumor cells and in situ cells, noteworthy research points include the identification of two distinct modes of tumor growth in bone metastases, the influence of type II pneumocyte on lung metastases, the paradoxical role of Kupffer cells in liver metastases, and the breakthrough of the blood-brain barrier (BBB) breach in brain metastases. Overall, this review provides a comprehensive overview of the characteristics of breast cancer metastases, shedding light on the pivotal roles of immune and resident cells in the development of distinct metastatic foci.

**Graphical Abstract:**

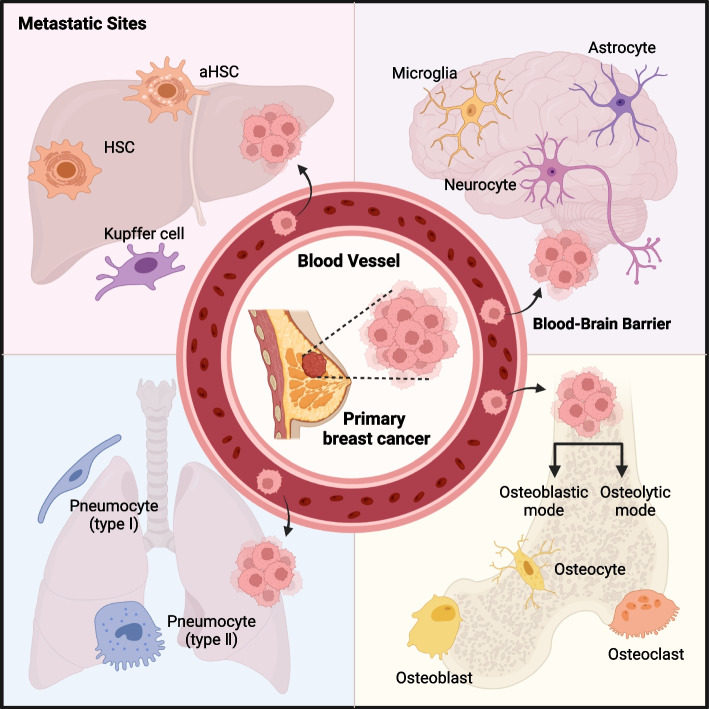

Video Abstract

**Supplementary Information:**

The online version contains supplementary material available at 10.1186/s12964-023-01418-4.

## Background

Breast cancer (BC) is the most common malignancy and second leading cause of cancer-related deaths among female population worldwide. According to the latest statistics, approximately 2.8 million new cases of invasive breast cancer and 51,400 instances of ductile carcinoma in situ (DCIS) arose in 2022, resulting in an estimated 43,250 fatalities [1]. It was reported that 50% of localized breast cancers ultimately metastasized [2]. Compared to those with no metastasis in breast cancer, patients with metastatic breast cancer (MBC) have a considerably worse prognosis, with a median survival time of 2–3 years and a mere 25% survival rate over 5 years [3].

During the metastasis process, a commonly endorsed theory is the “seed and soil” doctrine, in which metastatic breast tumor cells are considered as the “seeds”, while the “soil” symbolizes the microenvironment in the metastatic niche [4]. “Seeds” and “soil” undergo a bi-directional selection during transfer, resulting in different types of breast cancers demonstrating various propensities to metastasize to specific organs, a phenomenon termed “organotropism”. For instance, the luminal subtype exhibits a preference for bone metastasis, while triple-negative breast cancer (TNBC) generally tends toward visceral metastasis. Notably, regardless of the ultimate target, the initial stages of the cancer metastasis cascade share similarities across many types of cancer [5]. A prime example would be the epithelial mesenchymal transition (EMT) program, which plays a central role in the detachment of cancerous cells from the primary tumorand is regulated by transcription factors like Snail, Slug, and Twist. Most solid tumors are encapsulated by a complex network of the extracellular matrix (ECM), basement membrane and vascular system. Matrix metalloproteinases (MMPs) are critical to the degradation of the basement membrane and ECM, whereas the vascular system’s disruption and deterioration, often instigated by the tumor, assist cancerous cells in their entry into the bloodstream and subsequent spread to distant organs [6, 7].

While breast cancer subtypes offer crucial guidance for clinical treatment, accurate treatment for metastatic breast cancer patients requires an exhaustive understanding of the processes facilitating metastasis and the associated microenvironments. Traditional biological methodologies, including immunohistochemistry (IHC), immunofluorescence (IF), and nascent cytometry by time-of-flight (CyTOF), have demonstrated limited capabilities in targeting specific cellular populations, thus impeding comprehensive analysis of the considerably heterogeneous tumor microenvironment (TME) [8]. Nevertheless, swift advancements in technologies, such as single-cell sequencing and spatial transcriptomics, now give researchers the ability to extensively investigate and comprehend the tumor microenvironment.

In this review, we aim to provide an overview of microenvironmental heterogeneity in breast cancer metastases across various organ sites, demonstrating the complex interactions between cancer cells and microenvironmental cells in the environment as the cancer grows and spreads.

## The reciprocal adaptation of breast cancer metastasis and the surrounding microenvironment

The intricate interplay among the secreted protein profiles of malignant tumors and the immune and resident cells in tissues can potentially form a pre-metastatic niche in secondary organs, which, in turn, facilitates the homing of cancerous cells. The variations in this niche encompass four steps: (i) vascular changes including vascular permeability and the production of adhesion molecules, (ii) activation of matrix components and ECM remodeling, such as the secretion of MMPs, (iii) recruitment of immune cells, and (iv) alterations like metabolic adaptationin in resident cells [9, 10].

Following extravasation into the bloodstream, a mere 0.01% of circulating tumor cells (CTCs) effectively infiltrate and ultimately establish themselves in distant organs [11]. CTCs primarily disseminate to remote locations through blood circulation as well as vascular structure and adherence to endothelial cells. The survival, dormancy, or colonization of tumor cells in distant organs hinges on their surrounding environment established later [12].

The colonization stage is deemed the most intricate and constraining phase in the metastatic cascade, involving stress hormones, chemokines, and other factors [13]. The ensuing development of metastatic tumors is influenced by the presence of immune and stromal cells in the host tissue [14]. studies have revealed that the communication between cells in the tumor microenvironment (TME) and cancer cells evolves over time, aiding survival and proliferation of metastatic tumor cells. Notably, breast tumor cells initiating tumors secrete increased volumes of G-CSF due to heightened mTOR signaling, leading to a surge in myeloid-derived suppressor cells (MDSCs). Subsequent to MDSC removal, there is a boost in the frequency of tumor-initiating cells [15]. Owing to the heterogeneity of resident cells in various organs, intercellular communication exhibits divergent patterns across tissues (Table 1). Thereby, the ensuing sections will discuss each organ individually in our review.

**Table 1:**
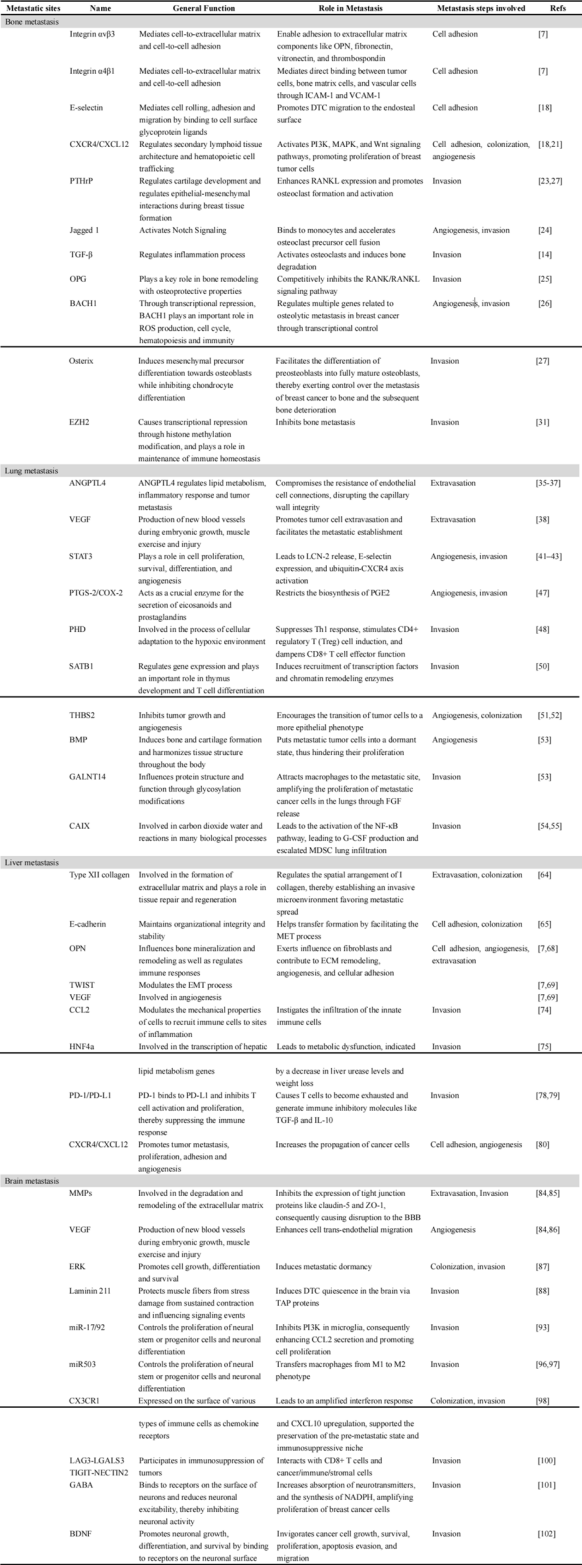
Molecules involved in different breast cancer metastasis

## Microenvironmental characteristics of breast cancer metastasis in different organs

### Bone metastasis

Bone is the most common site for breast cancer metastasis, with around 70% of breast cancer patients with metastasis experiencing distant bone metastasis during the disease progression [16]. The presence of advanced metastatic disease significantly impacts bone functionality, contributing to the rapid progression of cancer.

Dynamic interactions between resident cells such as osteoblasts, osteoclasts, and immune cells form a highly complex bone and bone marrow environment. Crucial elements of bone, such as osteoblasts, osteocytes, osteoclasts, and mesenchymal stem cells, are pivotal to the formation, maintenance, and support functions of bone. Furthermore, the bone marrow contains hematopoietic stem cells, diverse hematopoietic progenitor cells, and mature blood cells, all of which are responsible for hematopoietic activities [17]. Moreover, bones and bone marrow have an abundant blood flow. This vascular system, coupled with sensory and sympathetic nerves and their associate supportive cells, forms a complex network within the bone that sustains its structure and function [17].

The survival and colonization of of disseminated tumor cells (DTCs) within the bone metastatic microenvironment heavily rely on cell adhesion, which is facilitated by the interaction of various adhesion molecules. For instance, tumor cells express integrin αvβ3, enabling adhesion to extracellular matrix components like osteopontin (OPN), fibronectin, vitronectin, and thrombospondin. Other tumor cells express integrin α4β1, mediating direct binding between tumor cells, bone matrix cells, and vascular cells through intercellular adhesion molecule-1 (ICAM-1) and vascular cell adhesion molecule 1 (VCAM-1) [7]. The perivascular microenvironment plays a crucial role in supporting the primary settlement of DTCs within the bone marrow and influences their subsequent development. Price et al. Discovered that DTCs migration towards the endosteal surface of the bone is induced by endothelial cell-derived E-selectin, and DTCs retention within the perivascular ecological niche is mediated by CXCL12 [18]. Prior to developing into a macroscopic metastasis, tumor cells may remain dormant for several years as single cells or micrometastases. Hypoxia, inhibition of the PI3K-AKT pathway, and thrombospondin-1 (TSP-1) induced vascular inhibition influence metastatic dormancy. The NF-κB pathway in DTCs significantly promotes the transition from dormant to overt bone metastasis [7].

Bone metastasis is of two types based on ossification or bone resorption: osteolytic and osteoblastic. Osteolytic metastasis involves osteoclasts, causing local bone destruction and formation of lytic lesions commonly referred to as “punched out” lesions, whereas osteoblastic metastasis is characterized by an increased osteoblast activity resulting in dense bone sclerosis. In some cases, both processes can be expedited, leading to “mixed” lesions with both resorptive and sclerotic components [19].

The osteolytic bone metastasis mode, most common for breast cancer bone metastases, is distinguished by osteoclastic bone destruction induced by IL-11, coupled with promotion of tumor growth and osteoblast proliferation driven by angiogenesis through the connective tissue growth factor (CTGF) [20]. Metastasis progression is modulated by the interaction of CXCR4 with CXCL12 and the promotion of angiogenesis. MMP-1, which help faciliting cancer cell invasion, can be triggered by the RANK/RANKL signaling pathway by disintegrating collagen molecules in the extracellular matrix. This process prepares the endosteal surface for osteoclastic bone resorption culminating in bone destruction [21]. Tumor cells secrete several osteolytic factors, including PTH-related protein (PTHrP), IL-11, and Jagged 1, which contribute to bone degradation. PTHrP and IL-11 enhance RANKL expression, encouraging osteoclast formation and activation, while Jagged1 directly engages with monocytes to expedite osteoclast precursor cell fusion [22–24]. It has also been proven that transforming growth factor-β (TGF-β) advances tumor proliferation. Such growth factors are sequestered within osteoblasts and are subsequently released, thereby activating osteoclasts and inducing bone degradation [14]. Moreover, an abnormal MMP-1 manifestation may reduce the expression of osteoprotegerin (OPG), which is a bone-protective factor that competitively inhibits RANK/RANKL signaling pathway in osteoblasts [25].

As for the “seed” that serves as the primary regulatory factor in metastasis, the tumor cells, transcription factor BACH1 regulates multiple genes related to osteolytic metastasis in breast cancer through transcriptional control [26]. Furthermore, Osterix, a recently identified transcription factor, can promote the differentiation of pre-osteoblasts into fully mature osteoblasts. Osterix also modulates MMPs, vascular endothelial growth factor (VEGF), IL-8, and PTHrP to manage the dissemination of breast cancer to the bones and resultant bone loss [27].

In osteoblastic bone metastasis, tumor cells release diverse elements, including endothelin-1 (ET-1), growth differentiation factor 15 (GDF15), and bone morphogenetic protein (BMP) which stimulate the proliferation and differentiation of osteoblasts principally via the Wnt signaling pathway [28]. The addition of osteoblastic process intensifies the intricacy of osteolysis, often observed in diverse types of tumors. Research illustrates that the Wnt pathway plays a major role in the formation of bone metastasis in breast cancer by interacting with IL-1β and dictating the stemness of breast cancer cells [29]. Furthermore, a multitude of proteins specifically associated with bone metastasis have been identified by comparing the proteomics or secretome of bone metastasis derivatives and their parental cells. Included are inhibitors of tissue proteases, proteins that function with collagen, and activators of fibrinolysis [30].

**Fig. 1:**
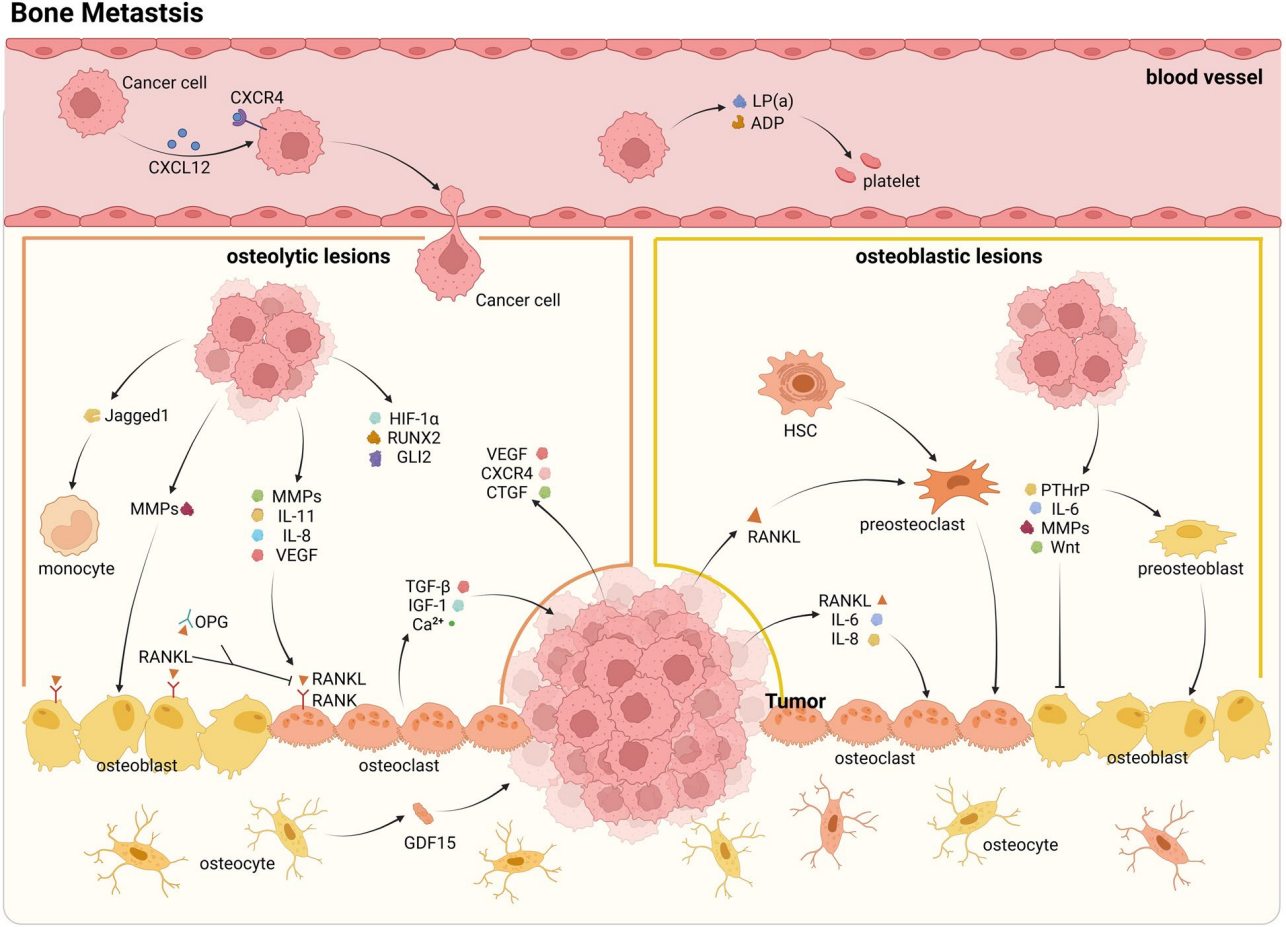
Cell-cell communication characteristics of bone metastasis in breast cancer

It is crucial to acknowledge that the formation of bone metastases may not represent the ultimate stage in the metastatic process. In actuality, a mere 6% of individuals with breast cancer experience the development of metastases in a single organ [17]. Research indicates that tumor cells that metastasize to bone tissue retain their capability to disseminate further. More specifically, the bone microenvironment triggers EZH2-driven epigenomic reprogramming, and targeted inhibition of this gene has the potential to impede subsequent metastasis in bone [31] (Fig. 1).

DTCs demonstrate enhanced recruitment and adhesion facilitated by the chemokine CXCL12 and platelets. In an osteolytic mode, DTCs secrete IL-8, IL-11, VEGF, and MMPs to activate the RANK/RANKL signaling pathway, while endothelial cells secrete OPG to competitively inhibit this process. Subsequently, domesticated osteoclasts produce TGF-β, IGF-1, and Ca^2+^ to facilitate metastatic growth. HIF-1α, RUNX2 and GLI2, which are produced by DTCs, can also remodel the bone environment for tumor growth. In the osteogenic mode, DTCs influence osteoblasts through PTHrP, IL-6, and Wnt signaling to promote bone growth, among them PTHrP also promotes preosteoblast differentiation. While RANKL secreted by tumor cells promotes preosteoclast differentiation. Additionally, osteoblasts secrete GDF-15 to further facilitate tumor development. Abbreviations: ADP, Adenosine diphosphate; CTGF, Connective tissue growth factor; DTC, Disseminated tumor cell; GDF, Growth differentiation factor; HIF, Hypoxia-inducible factor; HSC, Hepatic stellate cell; IGF-1, Insulin-like growth factor-1; IL, Interleukin; LP, Lipoprotein; MMP, Matrix metalloproteinaseo; OPG, Osteoprotegerin; RUNX, Runt-related transcription factor; TGF, Transforming growth factor; VEGF, Vascular endothelial growth factor.

### Lung metastasis

Approximately 15 to 20% of breast cancer patients develop lung metastases, the second most common type of breast cancer metastasis after bone. The metastases potentially disrupt regular pulmonary mechanisms, thereby prompting coughing, dyspnea, hemoptysis, and ultimate fatality. Considering the lack of common and effective therapeutic interventions, patients frequently confront an unfavorable prognosis, typically marked by a median survival rate approximating 15 months [32].

As CTCs travel through the lungs, they potentially interact with an expansive vascular system, encompassing up to 100 square meters. Considering that these tumor cells are on average five times larger than the particularly conservative pulmonary capillaries, it is highly likely that breast cancer cells may become lodged within these capillary beds, leading to eventual extravasation into the lung tissue [33]. In contrast to the non-continuous endothelial cells present in the skeletal vascular system, which can be easily traversed by other cells, the endothelial cells of the pulmonary capillaries are tightly interconnected. In order to invade and proliferate, breast cancer cells need to penetrate this robust barrier [34]. TGF-β, produced by stromal cells, macrophages, and cancer cells, induces the Smad signaling pathway, consequently activating ANGPTL4 [35]. ANGPTL4 then compromises the resistance of endothelial cell connections, disrupting the capillary wall integrity. This breach increases the permeability of pulmonary capillaries, allowing tumor cells to leak from blood vessels into lung tissue where they can establish and grow [36, 37]. At the same time, tumor cells secrete chemokines such as CCL2 in order to attract inflammatory monocytes, which subsequently facilitate the metastatic establishment of the tumor by secreting VEGF [38]. Tumor cell extravasation is further enabled by certain substances, including VEGF secreted by monocytes, as well as MMP and COX2 produced by tumor cells.

Notably, due to the exposure to external environment, the lung is vulnerable to different external stimuli that can potentially cause inflammation [39, 40]. Such inflammation caused by lipopolysaccharide (LPS) and cigarette smoke is strongly linked to breast cancer metastasis in the lungs. This is marked by an increase in neutrophil infiltration and the elevation of pro-inflammatory cytokines. The underlying mechanisms likely involve the activation of STAT3, leading to lipocalin-2 (LCN-2) release, E-selectin expression, and ubiquitin-CXCR4 axis activation [41–43].

Upon invasion into lung tissue, breast cancer cells rely on resident lung cells for colonization and metastatic growth. It is generally recognized that normal lung tissue contains alveolar and stromal macrophages [44]. During the initial phases of breast cancer cell infiltration into lung tissue, type II alveolar cells display stem cell traits and eventually differentiate into diverse lung cell types. Such creation of a favorable environment for the growth and spread of breast cancer cells leads to the development of a metastatic site [45].

As for immune cells, neutrophils adopt an anti-tumor phenotype during the initial phases of cancer metastasis. This phenotype is characterized by an absence of MMP, overexpression of pro-inflammatory and anti-cancer signaling pathways such as JAK-STAT, and an increase in interferon signaling (Irf9). However, as the tumor metastasis evolves, these neutrophils transition to a pro-cancer phenotype characterized by a decline in IL-1β and heightened expression of MMP-9 expression, along with curtailed T cell activation, decreased production of cytokines, and diminished interferon γ signaling. Similar patterns have been observed in monocytes [46]. Further research indicates that CD140a + mesenchymal cells in the lungs can alter neutrophil behaviour, endowing them with potent immunosuppressive capabilities. This is primarily attributed to the action of the enzyme Prostaglandin-endoperoxide synthase-2 (PTGS-2/COX-2) which restricts the biosynthesis of PGE2 [47]. Furthermore, the immune response against cancer cells is hampered by oxygen-sensitive prolyl hydroxylase (PHD) protein in T cells, which suppresses Th1 response, stimulates CD4+ regulatory T (Treg) cell induction, and dampens CD8+ T cell effector function. As a consequence, cancer cells begin to circulate and settle in the lungs [48].

Apart from the traditional signaling pathways like Myc and TGF-β, numerous novel controllers of lung metastasis have been identified. SATB1-directed recruitment of transcription factors and chromatin remodeling enzymes incite substantial transcription changes and are strongly linked to lung metastasis [49, 50] Thrombospondin-2 (THBS2), which is expressed in lung fibroblasts, encourages the transition of tumor cells to a more epithelial phenotype [51, 52]. Fibroblasts have also been found to secrete bone morphogenetic protein (BMP), hindering the proliferation of metastatic cancer cells. However, such inhibition can be reversed by the secretion of N-acetyl-galactosaminyltransferases 14 (GALNT14), which can revive dormant DTCs and promote ensuing metastatic growth. Also, tumor-derived GALNTs attract macrophages to the metastatic site, thereby amplifying the proliferation of metastatic cancer cells in the lungs through fibroblast growth factor (FGF) release [53].

**Fig. 2:**
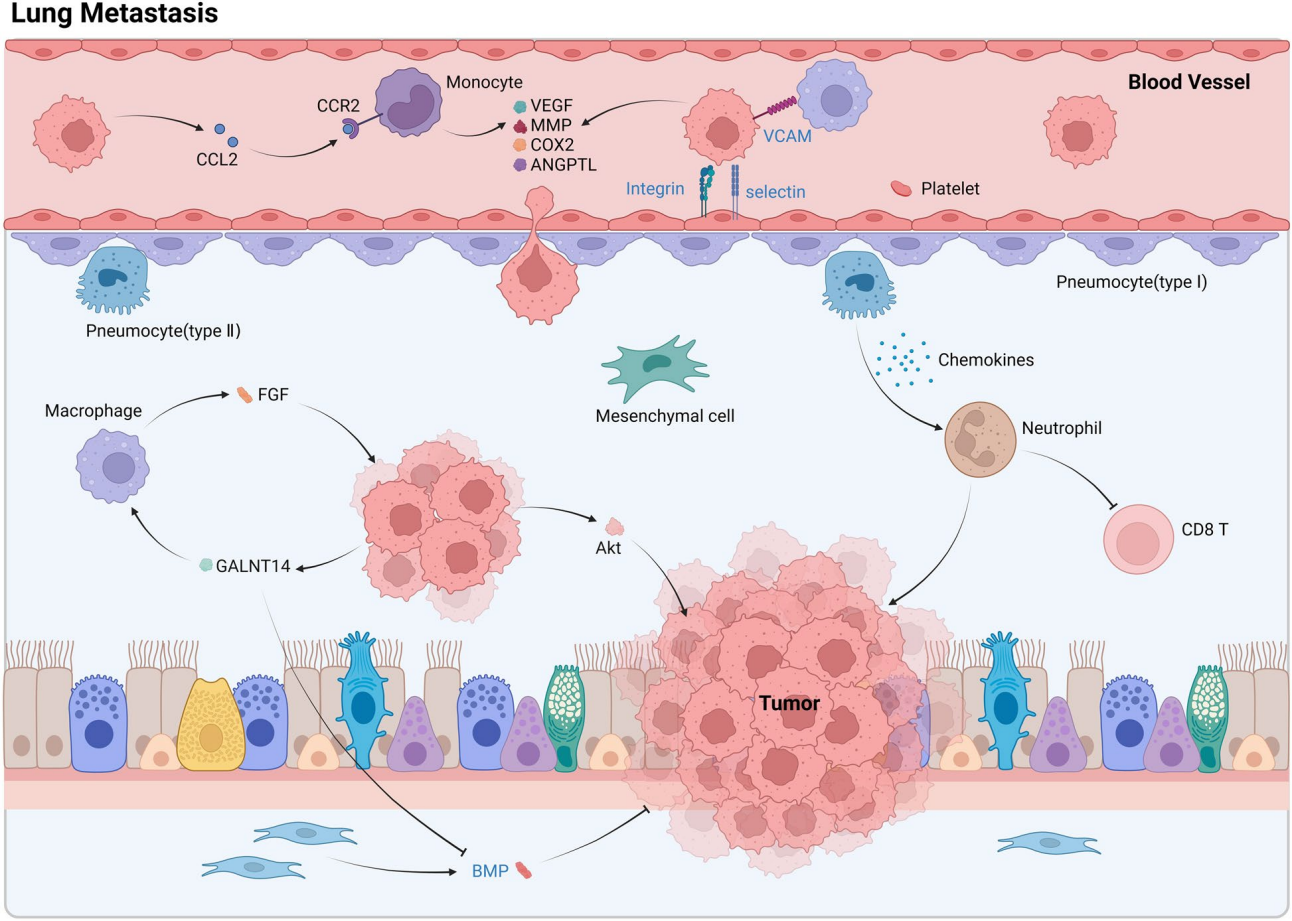
Cell-cell communication characteristics of lung metastasis in breast cancer

The hypoxia-induced activation of carbonic anhydrase IX (CAIX) has been discovered to aid in the production of renowned soluble agents of breast cancer metastasis, such as CCL5 and CXCL10. Additionally, CAIX in breast cancer cells leads to the activation of the NF-κB pathway, resulting in G-CSF production and increased MDSC lung infiltration. Furthermore, the advancement of breast cancer to the lungs is facilitated by the activation of MDSCs by innate lymphoid cells (ILCs) via the continuous activation of the IL-33/ST2 signal during the metastatic process, fostering tumor growth [54, 55]. The proliferation of metastatic tumors can also be facilitated by various other mechanisms, including increased collagen and elastin crosslinking, environmental changes from platelet-recruited myeloid cells, and chemokine release by type II alveolar cells to attract neutrophils [56] (Fig. 2).

Circulating tumor cells and recruited monocytes secrete VEGF, MMP, COX2, and ANGPTL, resulting in heightened vascular permeability. Additionally, integrins and selectins facilitate tumor cell adhesion, collectively facilitating tumor cell extravasation. Within lung tissue, type II alveolar epithelial cells release cytokines that modulate neutrophil activity, consequently suppressing CD8+ T cells and fostering tumor progression. Additionally, macrophages engage with tumor cells and modulate lung metastasis, this process proteins, and GALNT14 is able to regulate lung metastasis by affecting the expression of BMPs. Moreover, the presence of tumor-derived GALNTs serves to attract macrophages to the metastatic site, thereby amplifying the proliferation of metastatic cancer cells within the pulmonary region by means of FGF release. Abbreviations: BMP, Bone morphogenetic protein; COX, Cyclooxygenase; FGF, Fibroblast Growth Factor; GALNT, N-Acetylgalactosaminyltransferase; MMP, Matrix metalloproteinaseo; VCAM, Vascular cell adhesion molecule; VEGF, Vascular endothelial growth factor.

### Liver metastasis

Liver metastases rank second to lung metastases in terms of incidence, yet the survival rate is notably inferior compared to local recurrence, bone and lung metastasis. The expected 5-year overall survival rate stands at approximately 8.5% [57].

Liver metastasis is greatly influenced not only by the intracellular molecules of tumor cells but also by the hepatic organizational structure and microenvironment. These significantly contribute to the initial anchoring and eventual engraftment of breast cancer cells within the liver. Such tissue features, coupled with the dual blood supply from the portal vein and hepatic artery, provide a conducive setting for infiltration of CTCs leading to secondary liver metastases [58]. Additionally, constant exposure to various microorganisms and pathogens occurs as the liver is directly provided with blood from the gastrointestinal tract [59]. The remarkable components and response of antigen-presenting cells in the liver intricately balances its immune response to food-derived and microbial antigens versus its ability to respond to life-threatening pathogens [60].

Studies have identified a connection between the attachment of malignant cells to sinusoidal endothelial cells and the EMT process in human mammary neoplasms [61]. Research suggests that type IV collagen, along with the well-known role of type I collagen, can impact the transcriptional profile of cancer cells, promoting survival, invasion, and leading to epithelial-mesenchymal transition. These discoveries gives insight into the involvement of type IV collagen in tumor expansion [62, 63]. Tumor-associated fibroblasts (CAFs) secrete type XII collagen, which has the ability to regulate the spatial arrangement of I collagen, thereby establishing an invasive microenvironmentfavoring metastatic spread. Consequently, patients with elevated levels of type XII collagen have increased susceptibility to experiencing metastatic recurrence [64].

The loss of E-cadherins is a significant characteristic of EMT and is widely recognized as an invasive phenotype. Nonetheless, the upregulation of E-cadherin expression can contribute to the formation of liver metastases in breast cancer cells, potentially associated with the mesenchymal-epithelial transition (MET) at the metastatic site [65].

Other factors besides cadherins, including MMPs, integrins, and platelets, play a role in tumor cell adhesion and colonization. Specifically, the activation of cell proliferation and liver metastasis can be facilitated by N-cadherin and MMP-9, surpassing the suppressive effect of E-cadherin [66, 67]. Either direct interactions with receptors like VLA-4 or indirect adhesion and recruitment of platelets and neutrophils can promote cancer cell adhesion. In addition, tumor cells in hypoxic conditions enhance the transcription of a variety of genes such as OPN, TWIST, and VEGF [68, 69]. These proteins exert influence on fibroblasts and contribute to ECM remodeling, angiogenesis, and cellular adhesion. Collectively, these factors create an advantageous microenvironment conducive to the infiltration and dissemination of tumor cells [7].

The survival and proliferation of metastatic cells in the liver depend on the intricate interactions among tumor cells and various local resident subpopulations, namely sinusoidal endothelial, stellate, and Kupffer cells [70]. Upon reaching the liver, Kupffer cells use specific receptors like Dectin-2 to identify and eradicate invading tumor cells, thus safeguarding the liver from metastasis [71]. Moreover, tumor cell elimination can be further facilitated by Kupffer cells and liver sinusoidal endothelial cells through the secretion of tumor necrosis factor (TNF), nitric oxide (NO), and reactive oxygen species (ROS), or via cell apoptosis mediated by the perforin-granzyme pathway or Fas-FasL pathway initiated by liver natural killer cells [72, 73]. As a result of tumor cell death and tissue damage, various cytokines including IL-1, IL-6, IL-8, IL-12, and IL-18, as well as the chemokine CCL5, are subsequently released [74]. These local inflammatory responses can further triger the chemokines such as CCL2, thus instigating the infiltration of the innate immune cells. Moreover, Research have revealed that communication between the immune system and hepatocytes occurs via IL-6-STAT3, leading to HNF4a loss and subsequent metabolic dysfunction, indicated by a decrease in liver urease levels and weight loss [75].

Intriguingly, while Kupffer cells primarily exhibit antitumor properties during early metastasis, they can enhance metastasis and early colony formation by secreting growth factors such as PDGF and VEGF following extravasation and early colony formation, which in turn stimulate cancer cell proliferation and angiogenesis [70]. Simultaneously, Kupffer cells have the ability to attract other cells with pro-metastatic functions such as neutrophils, monocytes, macrophages, and MDSCs, which can ultimately lead to the formation of an immunosuppressive microenvironment [76].

**Fig. 3:**
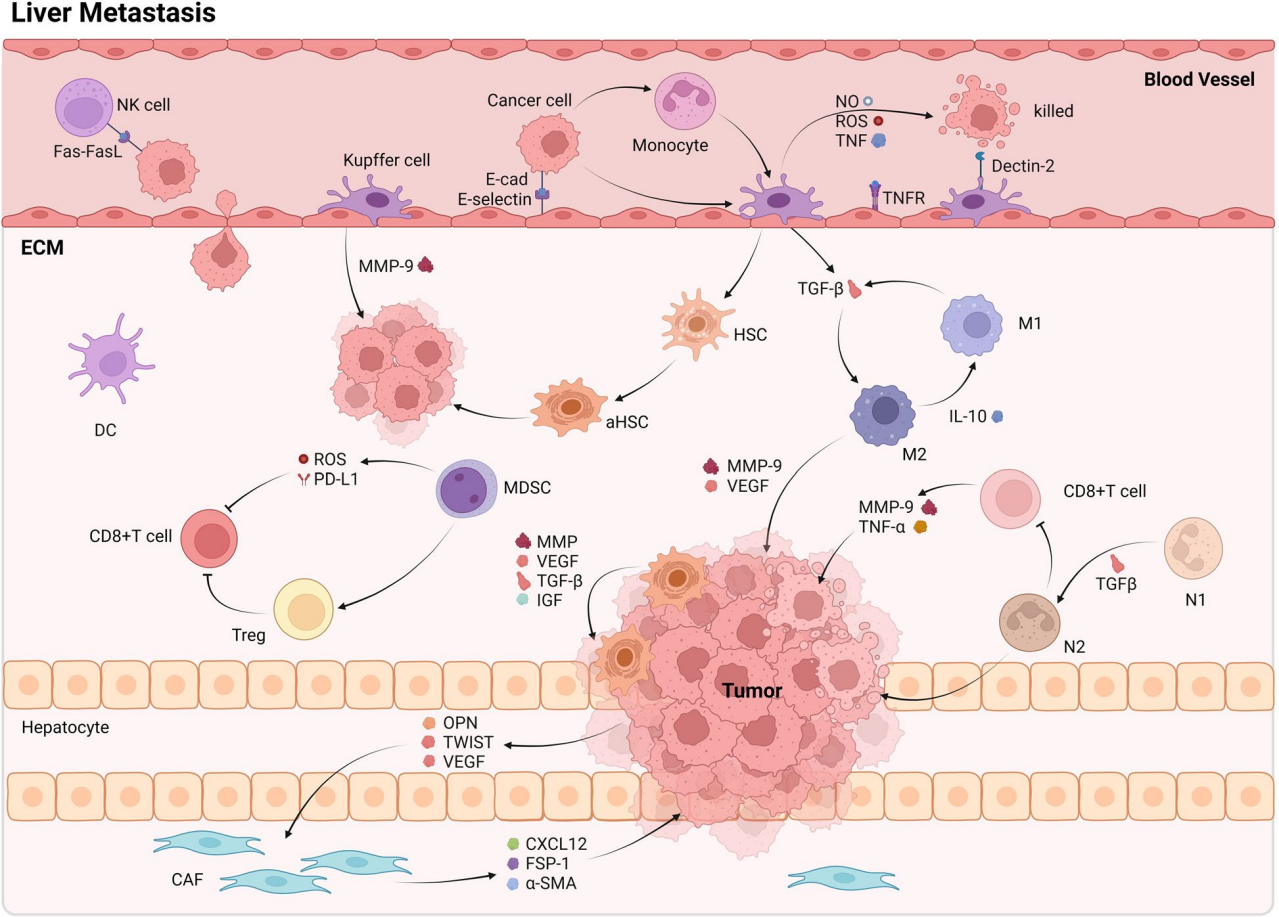
Cell-cell communication characteristics of liver metastasis in breast cancer

The establishment and proliferation of metastatic cancer cells within the liver also prompt the release of TGF-β, thereby further promoting tumor expansion [77]. Sinusoidal endothelial cells, acting as resident liver cells with antigen-presenting functions, can encourage immune tolerance by expressing PD-1 and binding with PD-L1 on T cells, leading T cells to become exhausted and generate immune inhibitory molecules, like TGF-β and IL-10 [78, 79]. Hepatic stellate cells (HSCs), stimulated by inflammatory signals released by tissue-resident fibroblasts or macrophages, lead to the secretion of CXCL12, promoting the endurance and multiplication of breast cancer when attached to CXCR4 on malignant cells, thereby enabling the increased propagation of cancer cells [80]. Furthermore, the activation of hepatic stellate cells (HSCs) elicits an increased accumulation of extracellular matrix, leading to hepatic fibrosis progression. This fibrotic environment impedes the infiltration of anti-tumor immune cells, providing a favorable environment for metastatic tumor growth (Fig. 3).

Breast cancer cells release adhesion molecules, such as E-cadherin, which facilitate the attachment of CTCs to endothelial cells. Monocytes stimulate Kupffer cells, leading to the production of NO, ROS, and TNF, which collectively contribute to the elimination of CTCs. Kupffer cells also activate HSCs within the tissues, prompting the secretion of MMP, VEGF, TGF-β, and IGF. These factors act on HSCs themselves, promoting tumor progression. Additionally, Kupffer cells facilitate the differentiation of macrophages into the M2 phenotype through TGF-β signaling. N2 type neutrophils impede the tumor-killing capabilities of CD8+ T cells and shape an immunosuppressive microenvironment. Metastatic foci have the potential to impact CAF through the action of OPN, TWIST, and VEGF, resulting in the secretion of CXCL12, FSP-1, andα-SMA to facilitate tumor growth. In addition, MDSC exerts an immunosuppressive function by activating Treg and suppressing CD8+ T cells. Abbreviations: CAF, Cancer associated fibroblast; DC, Dendritic cell; FSP, Fibroblast specific protein; IGF, Insulin-like growth factor; MDSC, Myeloid-derived suppressive cell; MMP, Matrix metalloproteinaseo; NO, Nitric Oxide; OPN, Osteopontin; ROS, Reactive oxygen species; TGF, Transforming growth factor; TNF, Tumor Necrosis Factor; Treg, Regulatory T cell; TWIST, Twist family bHLH transcription factor; VEGF, Vascular endothelial growth factor; SMA, Smooth muscle actin.

### Brain metastasis

Approximately 10–15% of breast cancer patients develop cerebral metastases, one of the most severe types of metastases. This ailment is associated with a poor prognosis, as the chance of surviving for one year is only 20% [81].

Breast cancer brain metastasis primarily occurs when breast cancer cells are able to penetrate the blood-brain barrier (BBB) [82]. Intercellular communication, tight junctions, adheren junctions, and gap junctions are utilized by brain microvascular endothelial cells to create a strong barrier [83]. However, Breast cancer cells can infiltrate this protective layer of the brain by increasing the permeability of the BBB as well as the penetration ability of breast cancer cells. This is accomplished through diverse mechanisms, including the secretion of multiple cytokines such as VEGF and MMPs that alter the permeability of the BBB [84]. Tumor cells have the capacity to generate MMP-2 and MMP-9, which inhibit the expression of tight junction proteins like claudin-5 and ZO-1, consequently causing disruption to the BBB [85]. VEGF significantly promotes angiogenesis and noticeably rises in response to integrin stimulation. This process disrupts the proteins of the tight junction and adhesion links within the endothelium, which subsequently augments vascular permeability [86]. Furthermore, these newly formed blood vessels concomitantly enhance the growth of metastases.

When DTCs cross the BBB, the majority of them undergo apoptosis due to the robust anti-tumor immune response. However, some DTCs can enter a dormant state with potential for reactivation, while others may enter a proliferative state. The activation of the p38 signaling pathway blocks the activity of extracellular signal-regulated kinase (ERK), thereby inducing metastatic dormancy [87]. Studies by Ghajar et al. reveal that the presence of laminin 211 in astrocytes plays a crucial role in inducing a state of quiescence in DTCs within the brain, mediated via dystrophin glycans trapping Yes-associated protein (YAP) within the cytoplasm [88]. Yet, the presence of TGF-β and OPN can stimulate the reawakening of dormant cells, leading to metastatic recurrence [89]. Another interesting phenomenon is that active metastatic cells have a propensity to generate micrometastases adjacent to blood vessels via vascular co-option, which prepares metastatic lesions for continuous growth. Specifically, tumor cells adhere to capillary surfaces and stretch, forming elongated clusters or capillary rings around the vessels. Lack of vascular co-option may result in metastatic failure [90].

Tumor cells that enter a proliferative state initiate interactions with reactive astrocytes or microglia, activating various signaling pathways that facilitate metastatic growth, disease progression, and evasion of the immune response. Reactive astrocytes can limit DTCs count by activating fibrinolysis. Nonetheless, malignant cells can produce inhibitors of serine protease that impede the initiation of fibrinolysis and augment DTC accumulation. Excessive production of the mTOR serine/threonine kinasecan lead to microglia attraction, tumor formation, and primary T cells death within the tumor microenvironment [91]. Interestingly, reactive astrocytes secrete several inflammatory agents such as IL6, IL8, TNF-α, and IFN-α that can promote the cell movement and infiltration [92]. Moreover, astrocyte-derived extracellular vesicles that convey miR-17/92 gene locus payload can suppress the PTEN, a PI3K inhibitor in microglia, consequently enhancing CCL2 secretion and promoting cell proliferation [93]. Finally, astrocytes can elicit immunosuppression through the suppression of CD8+ T cells and the polarization of TAMs into anti-inflammatory M2 macrophages in a STAT3-dependent manner [94, 95].

Microglia, known as tissue-resident macrophages in the brain, has a close interaction with DTCs. Similar to other macrophage populations, microglia possesses both anti- and pro-tumor properties. Activated microglia can produce a substantial quantity of molecules capable of mitigating or inducing inflammation, thereby impacting tumor progression and the formation of brain metastases. The M2 microglia phenotype is known to disrupt the central nervous system homeostasis and augments metastatic progression. The transition from M1 to M2 phenotype is induced by metastatic tumor cells utilizing the extracellular vesicle miR503, which facilitates tumor growth. However, the means through which these cells bypass the cytotoxic effects of M1 microglia remains ambiguous [96, 97]. Guldner et al. delved into the tumor microenvironment to investigate the response of bone marrow of the central nervous system during the advancement of brain metastasis. Their results highlighted the crucial role of microglia, denoted by a decrease in steady-state markers such as CX3CR1. This occurrence led to an amplified interferon response and CXCL10 upregulation, which supported the preservation of the pre-metastatic state and immunosuppressive niche [98].

Furthermore, the brain microenvironment can be significantly enhanced by tumor cells through the stimulation of growth factor receptors and activation of the AKT/PI3K/mTOR, MAPK, and NF-κB signaling pathways [99]. Through the use of single-cell sequencing analysis on metastatic lesions, Zou et al. discovered that the immune checkpoint molecules LAG3-LGALS3 and TIGIT-NECTIN2, which interact with CD8+ T cells and cancer, immune, and stromal cells, are the main contributors to immune evasion [100].

**Fig. 4:**
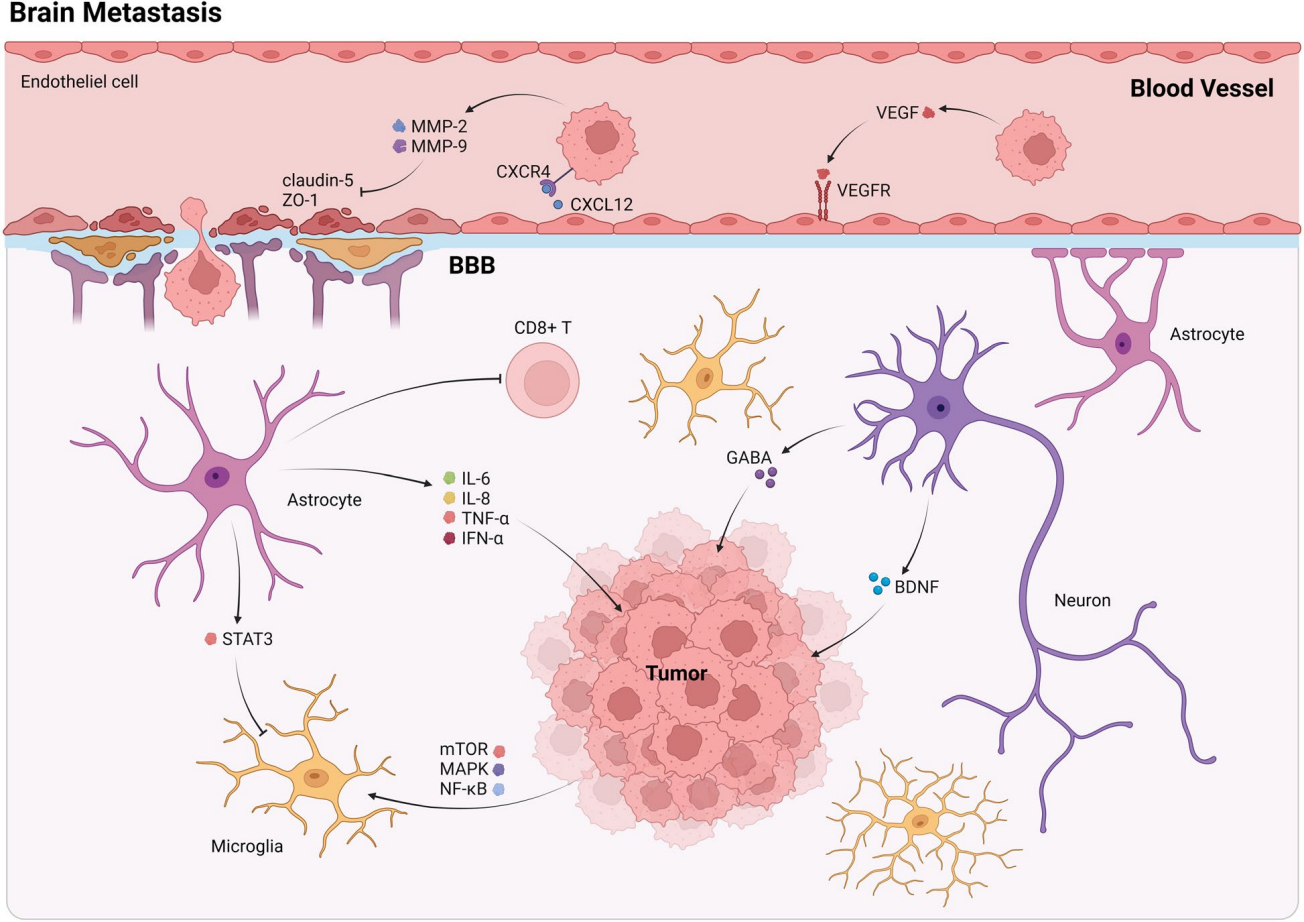
Cell-cell communication characteristics of brain metastasis in breast cancer

Endogenously generated neurochemicals that facilitate neuronal communication also play a role in transmitting information between neurons and propagating breast cancer to the brain. Previous studies have shown that increased expression of GABA transporters and receptors in brain metastases leads to greater absorption of neurotransmitters, increased synthesis of NADPH, and amplified proliferation of breast cancer cells [101]. Brain-derived neurotrophic factor (BDNF), a part of the neurotrophic factors, possesses the capacity to invigorate cancer cell growth, survival, proliferation, apoptosis evasion, and migration. The involvement of multiple signaling pathways including PI3K/Akt, Jak/STAT, NF-kB, and EGFR transactivation, are integral to the effectiveness of this mechanism [102] (Fig. 4).

During brain metastasis, CTCs act with vascular endothelial cells through CXCR4, MMP, and VEGF. Among them, MMP-2 and MMP-9 blocked the expression of tight junction proteins claudin-5 and ZO-1, thus helping tumor cells to cross the BBB. In brain tissue, astrocytes secrete various inflammatory factors such as IL6, IL8, TNF-α, and IFN-α on the one hand, and on the other hand, they can inhibit CD8+ T cells to exert immunosuppressive effects. Microglia can be affected by astrocyte-secreted STAT3 as well as tumor-secreted mTOR, MAPK, and NFκB. In addition, increases in the neurotransmitter GABA, and the neurotrophic factor BDNF can enhance the survival and proliferation of breast cancer cells. Abbreviations: BDNF, Brain-derived neurotrophic factor; GABA, Gamma aminobutyric acid; IFN-α, Interferon alpha; IL, Interleukin; MAPK, Mitogen activated protein kinase; MMP, Matrix metalloproteinaseo; mTOR, Mammalian target of rapamycin; STAT3, Signal Transducer and Activator of Transcription 3; TGF-β, Transforming growth factor beta; VEGF, Vascular endothelial growth factor; VEGFR, Vascular endothelial growth factor receptor; ZO-1, Zonula Occludens Protein-1;

## Conclusions and future perspectives

Overall, the propensity of breast cancer to metastasize is affected by a complex web of interrelated elements, including different cellular interactions and the tumor microenvironment. It is noteworthy that the composition of the metastatic microenvironment can vary across different metastatic locations, with certain constituents exerting a predominant influence on tumor progression.

For complicated breast cancer metastasis, researchers are coming up with methods for early diagnosis and intervention. Curently, there exists insufficient clinical tools for the detection of MBC. However, the liquid biopsy of various bodily fluids has recently emerged as a minimally invasive approach that offers real-time information on tumor biomarkers. This technique enables early diagnosis, as well as active monitoring of disease progression and recurrence following treatment. Currently proposed analytical priorities for liquid biopsies include CTC, circulating tumor DNA (ctDNA), tumoureducated platelets and extracellular vesicles [103].

The guidelines released online by ASCO in 2022 also updates 11 recommendations, and modifies the testing of MBC biomarkers in response to data from current practice. Among the primary biological markers discussed arect: phosphatidylinositol-4,5-bisphosphate 3-kinase catalytic subunit alpha (PIK3CA), deficient mismatch repair/microsatellite instability (dMMR/MSI), tumor mutational burden (TMB), neurotrophic tyrosine receptor kinase (NTRK), as well as ctDNA and CTCs [104].

Given the above, enhancing our comprehension of the tumor microenvironment and its constituents can offer significant insights for developing innovative therapeutic approaches that specifically address the microenvironment, thereby improving the clinical outcomes for breast cancer patients. Future research efforts should focus on elucidating the more precise roles of various microenvironmental components in different metastatic sites and identifying potential diagnostic as well as therapeutic targets to effectively tackle breast cancer metastasis.

## Data Availability

Not applicable.

## Abbreviations

BBB: Blood-brain Barrier
BC: Breast Cancer
BMP: Bone morphogenetic protein
CAIX: Carbonic anhydrase IX
CTC: Circulating tumor cell
DTC: Disseminated tumor cell
ECM: Extracellular matrix
EMT: Epithelial-mesenchymal transition
FGF: Fibroblast Growth Factor
GALNT: Polypeptide N-Acetylgalactosaminyltransferase
HSC: Hepatic stellate cell
IL: Interleukin
MBC: Metastatic breast cancer
MDSC: Myeloid-derived suppressor cell
MMP: Matrix metalloproteinaseo
OPG: Osteoprotegerin
Osx: Osterix
PTHrP: PTH-related protein
TNF: Tumor Necrosis Factor
TGF: Transforming growth factor
TME: Tumor microenvironment
VEGF: Vascular endothelial growth factor
